# Ethanolic Extract of Dried Leaves from the *Cerrado* Biome Increases the Cryotolerance of Bovine Embryos Produced *In Vitro*

**DOI:** 10.1155/2020/6046013

**Published:** 2020-11-22

**Authors:** Andrei Antonioni Guedes Fidelis, Gabriela de Oliveira Fernandes, Franscislete Rodrigues Melo, Ligiane de Oliveira Leme, Paulo Roberto Adona, Taynan Stonoga Kawamoto, Margot Alves Nunes Dode

**Affiliations:** ^1^Veterinary Department, University Center of Brasília-UniCeub, Brasília, Brazil; ^2^School of Agriculture and Veterinary Medicine, University of Brasília, Brazil; ^3^Laboratory of Animal Reproduction, Embrapa Genetic Resources and Biotechnology, Brasília, Brazil; ^4^University North of Paraná, Londrina, Brazil

## Abstract

*In vitro* embryo production (IVP) induces excessive production of reactive oxygen species (ROS), which affects blastocyst quality. Therefore, the supplementation of culture media with antioxidants is an alternative to overcome oxidative stress damage. However, there is a growing demand for the use of antioxidant compounds that are more natural and less toxic in cell cultures. The present study is aimed at evaluating the effect of ethanolic extracts from *cerrado* leaves on IVP. First, the antioxidant capacity and the amount of phenolic compounds of the leaves were evaluated. Then, the best ethanolic extract concentration composed of cagaita (*Eugenia dysenterica*) and murici (*Byrsonima crassifolia*) to be used during the *in vitro* culture of *in vitro*-produced embryos was determined. Afterward, we evaluated the influence of the extract of both plants on ROS and glutathione (GSH) production, while also evaluating the apoptosis and ROS metabolism gene expression. In a subsequent step, the effect of the ethanolic extracts of dried cagaita and murici leaves during embryonic cultivation on the cryotolerance of expanded blastocysts was studied. The results showed a significant reduction in the proportion of apoptotic cells from embryos cultivated with 0.01 mg/mL of the cagaita ethanolic extract, besides inducing an increase in the GPX4 and PRDX3 transcription levels. The murici ethanolic extract induced an increase in the transcription abundance of these genes but did not reduce the proportion of apoptotic cells. In addition, expanded blastocysts cultivated with extracts at a concentration of 0.01 mg/mL and cryopreserved had higher hatching rates and lower degeneration rates when compared to the frozen group previously supplemented with the extracts. Moreover, the apoptosis rate of embryos cultured for 12 h after cryopreservation was lower in groups previously exposed to extracts during *in vitro* cultivation. Such extracts may be used as alternatives to increase the cryotolerance of *in vitro*-produced embryos.

## 1. Introduction

*In vitro* embryo production (IVP) in cattle is a means for the rapid multiplication of individuals of high genetic merit in commercial systems. However, the *in vitro* culture system does not have optimal development conditions, and embryos are usually of lower quality than those produced *in vivo*. It is well established that embryos require energy for their rapid growth and that the generation of this energy includes the production of reactive oxygen species (ROS). Therefore, to ensure that the initial embryonic development takes place accordingly, a balance between the generation and the elimination of ROS, which is essential for intracellular signal transduction, should be maintained [1, 2]. Although certain ROS levels are necessary for the development of gametes and fertilization [3, 4], high ROS concentrations have been associated with a reduction in embryo viability. The main reason for this is that ROS damages biomolecules (oxidation of carbohydrates and proteins, fragmentation of nucleic acids, and lipid peroxidation) and induces blastomere apoptosis [5–7].

In the *in vivo* environment, embryos are constantly supplied with adequate nutrients, hormones, and growth factors. In addition, the epithelium of the genital tract is in charge of promoting the removal of metabolites produced by the embryo, thus ensuring a healthy environment that meets the embryo's needs. This scenario is very contrasting to the *in vitro* culture environment; there, the atmospheric concentration of oxygen used (20%), the excessive light exposure, the absence of antioxidant enzymes of the genital tract, the use of large volumes of culture media, and the static nature of the culture cause an accumulation of ROS and toxins in the medium, which affects the quality and cryotolerance of embryos produced *in vitro* [8–10].

Considering that the *in vitro* environment is very stressful for the embryo, supplementing the IVP medium with antioxidant compounds, which control the excessive production of ROS, can be very beneficial. In fact, some studies have shown that the supplementation of maturation and/or embryonic culture media with antioxidant compounds, such as cysteine, cysteamine, *β*-mercaptoethanol, and cystine, promotes an increase in embryo production and quality [11–14]. However, conflicting results on the toxicity of these compounds in the *in vitro* embryonic development of different species are still being described [12, 15, 16]. Thus, the demand for natural and efficient antioxidants, with relatively safe and nontoxic properties, is growing among different research groups.

Thus, phenolic compounds derived from the secondary metabolism of plants would be an interesting alternative as they have antioxidant characteristics and other medicinal properties, such as natural anti-inflammatory and antibiotic agents. Indeed, resveratrol, icariin, quercetin, anthocyanin, and other phenolic compounds have already been tested in the *in vitro* production of mammalian embryos and resulted in an increase in embryonic quality [17–26]. These suggest that plant extracts can be used to improve IVP results, owing their beneficial effects not only to their antioxidant activity but also to their additional beneficial properties. The Brazilian *cerrado* is the second largest biome in South America and the richest savanna in the world, with great biological diversity. In addition, this biome area has a peculiar climate and is often affected by intense fires. Several researchers [27–30] have reported that plants adapted to that soil and to the adverse weather conditions may have developed efficient molecular defense mechanisms against ROS, which involve the production of potent antioxidant compounds such as catechin, quercetin, epicatechin, ellagic acid, anthocyanins, and carotenoids, among other bioactive substances [28, 31]. However, there are no reports of these leaf extracts on *in vitro* embryo production.

Considering the great antioxidant potential of plants from the *cerrado* biome and the need to test new compounds for oxidative stress control in the IVP routine, the present study evaluated the effect of supplementing culture media with ethanolic extracts from dried leaves of *cerrado* plants on the quality and cryotolerance of bovine embryos.

## 2. Materials and Methods

Unless otherwise indicated, all reagents used were purchased from Sigma-Aldrich (St. Louis, MO, USA).

### 2.1. Preparation of Ethanolic Extracts from *Cerrado* Native Plants

Five plants were chosen to obtain leaf extracts: cashew (*Anacardium humile*), cagaita (*Eugenia dysenterica*), araticum (*Annona montana*), murici (*Byrsonima crassifólia*), and jenipapo (*Genipa americana*). The chosen plants are part of a plant bank in the laboratory for studies of antihelminthic, anti-inflammatory, and antimicrobial activities (University Center of Brasília, Brazil). The leaves, from adult trees, were collected in Planaltina, Federal District, Brazil (latitude 15°34′S and longitude 47°43′W), in winter (dry season) and were dehydrated and crushed in a 1 mm granulometry mill. The obtained powder was placed in a beaker that contained 99.3% ethanol PA (pro analysi) in a ratio of 1 : 5 weight/volume. The maceration was then carried out at room temperature under constant stirring for seven days and then filtered. The extract was thereafter concentrated using thermal blankets regulated at a temperature of 50°C and dried. Each mg of the dried prepared extract was diluted in 1 mL of NaCl 0.9%, reaching a concentration of 1 mg/mL. The extract was used for polyphenol dosing by the Folin-Ciocalteu method. The quantitative evaluation of the antioxidant activity was determined by an assay using the free radical capture method: ABTS (2,2-azino-bis (ethylbenzothiazoline-6-sulfonic acid)).

#### 2.1.1. Antioxidant Activity by the Free Radical Capture Method: ABTS

**Figure 1:**
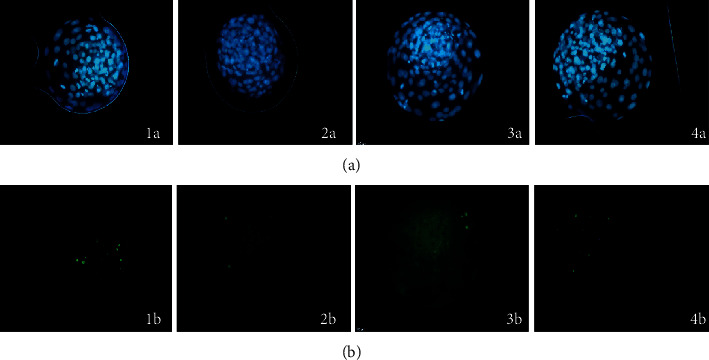
(a) Total cell number and (b) apoptotic cells in expanded blastocysts in the presence of different ethanolic extract concentrations of dry cagaita leaves: (1) control (0 mg/mL), (2) 0.01 mg/mL, (3) 0.1 mg/mL, and (4) 1 mg/mL.

The quantitative evaluation of the antioxidant activity was performed by monitoring the scavenging of the cation ABTS^∙+^ by the samples, by measuring the decrease in absorbance of solutions of different concentrations. This method is based on the generation of ABTS^∙+^by means of the reaction of ABTS with potassium persulfate, which has a maximum absorption at 734 nm. With the addition of an antioxidant, there is a reduction of ABTS^+^′ in the ABTS, and consequently, there is a loss of staining in the reactional medium. With the loss of color, the percentage of inhibition of ABTS^∙+^ is determined on the basis of a Trolox standard, subjected to the same conditions of antioxidant analysis [32]. In short, the lower the value of absorbance measured by a spectrophotometer, the greater the antioxidant activity. Each extract was diluted in a saline solution (five different concentrations: 50, 200, 300, 400, and 500 g/L). Thirty-microliter aliquots were placed in tubes with 3 mL of radical ABTS^∙+^, homogenized, and read in a spectrophotometer (UV-1800, Shimadzu, Nakagyo/Kyoto, Japan) at 734 nm, after six minutes. All analyses were carried out in triplicate. A Trolox standard curve of absorbance (Figure 1—Supplementary files) was determined at five concentrations (100, 500, 1000, 1500, and 2000 *μ*M). From the equation generated and the Trolox equivalent, the antioxidant capacity of the extracts was measured and expressed in *μ*M Trolox/g.

#### 2.1.2. Determination of Phenolic Compounds by the Folin-Ciocalteu Method

The phenolic compound content in the extracts was determined by using the Folin-Ciocalteu reagent (Merck, Darmstadt, Germany) as described by Singleton et al. [33]. The Folin solution was prepared using the Folin-Ciocalteu reagent and deionized water 1 : 1 (*v*/*v*). Then, 30 *μ*L of the ethanolic extract (1 mg/mL) and 75 *μ*L of the Folin solution were added in an Eppendorf tube (Eppendorf, São Paulo, Brazil). After 5 min of reaction, 75 *μ*L of a sodium carbonate solution (20%) and deionized water were added until a final volume of 600 *μ*L was reached. The solution was kept for 30 min, and then it was read in a spectrophotometer (UV-1800, Shimadzu®) at 750 nm. Gallic acid was used as the default. The results were expressed in mg of gallic acid equivalent (GAE) per 100 g of extract. All analyses were carried out in triplicate.

### 2.2. *In Vitro* Embryo Production

Cumulus-oocyte complexes (COCs) were obtained from slaughterhouse ovaries, transported to the laboratory in a saline solution (NaCl 0.9%), and supplemented with amikacin (250 *μ*g/mL) at 35°C. Follicles of 3 to 8 mm were aspirated using a syringe and an 18-gauge needle. The oocytes were selected according to their appearance, the number of cumulus cell layers, and the homogeneity of their cytoplasm. Only those classified as grades I and II were used [34]. The time between the beginning of the aspiration of slaughterhouse ovaries until the selection and maturation of oocytes was 1 h.

For each step of the IVP, 150 *μ*L of a specific medium (maturation, fertilization, or culture) was covered with mineral oil and incubated in an atmosphere of 5% CO_2_ and air at a temperature of 38.5°C.

For *in vitro* maturation (IVM), groups of 25 to 30 COCs were cultured in an *in vitro* maturation medium for 22-24 h. The IVM medium consisted of TCM 199 Earle's salts (Gibco BRL, Burlington, ON, Canada) supplemented with 10% fetal bovine serum (FBS, Gibco), 10 *μ*g/mL of follicle-stimulating hormone (FSH), 1 *μ*g/mL of L-glutamine, and 250 mg/mL amikacin sulfate.

After IVM, the COCs were fertilized with frozen semen previously tested for IVP. Motile spermatozoa were selected by the Percoll (GE Healthcare, Piscataway, NJ, USA) gradient (90% : 45%), as described by Machado et al. [35]. The inseminating dose was adjusted to a final concentration of 1 × 10^6^ sperms/mL. The gametes were coincubated for 12 h in a fertilization medium, which consisted of Tyrode's albumin lactate pyruvate supplemented with 2 mM penicillamine, 1 mM hypotaurine, 250 mM epinephrine, and 10 *μ*g/mL heparin [36]. After the *in vitro* fertilization (IVF) period ended, the presumptive zygotes were washed and transferred to the *in vitro* culture (IVC) with the synthetic oviduct fluid (SOF) culture medium supplemented with essential and nonessential amino acids, 0.34 mM trisodium citrate, 2.77 mM myoinositol, and 5% FBS [37], cocultured with cumulus cells. In the group cultured in a low O_2_ tension system (experiments 3 and 4), the presumptive zygotes were stripped off the cumulus cells with the aid of a vortex (Scientific Industries, NY, USA), in a washing medium for 1 min at 10,000 rpm. The washing medium was composed of TCM 199 with Hank's salts (Gibco) supplemented with 1% FBS and 250 mg/mL amikacin sulfate. Immediately after the removal of cumulus cells, they were transferred to the SOF medium and placed in an incubator at low O_2_ tension, where they were kept for seven days.

The insemination time was considered day (D) 0 (zero). Cleavage was evaluated on D2 (48 h postinsemination (pi)), and blastocyst rates were evaluated on D6 (144 h pi) and D7 (168 h pi) of the culture.

### 2.3. Total and Apoptotic Cell Numbers

The total and apoptotic cell numbers were determined using the TUNEL (terminal deoxynucleotidyl transferase dUTP nick end labeling) method (Thermo Fisher, Waltham, MA, USA), according to the manufacturer's instructions. Briefly, after three washes in PBS with 0.1% bovine serum albumin (PBS-BSA) medium, only expanded blastocysts (BX) of each group were incubated in freshly prepared 3.7% paraformaldehyde in PBS-BSA for 15 minutes. After washing twice in the PBS-BSA medium, embryos were incubated in freshly prepared 0.5% Triton X-100 in PBS-BSA for 20 minutes to allow partial permeabilization and immediately washed twice in PBS-BSA. After the completion of the TUNEL assay, the BX embryos were stained with Hoechst 33342, for 15 minutes. The fixed and stained embryos were washed three times in PBS-BSA, mounted onto a glass microscope slide, and analyzed under an epifluorescence microscope (Axioplan 2, Zeiss, Jena/Stuttgart, Germany). The results were expressed as a proportion of apoptotic cells/total cell number.

### 2.4. Measurement of ROS and Glutathione (GSH) Content

The intracellular ROS levels were quantified using 2,6-dichlorodihydrofluorescein diacetate (H_2_DCFDA) (Thermo Fisher, Waltham, MA, USA), and GSH levels were quantified with the fluorescent marker CellTracker Blue-4-chloromethyl-6,8-difluoro-7-hydroxycoumarin (Thermo Fisher), according to the manufacturer's recommendations. For the ROS evaluation, the expanded blastocysts obtained from all groups were stained and assessed by confocal microscopy. All images were captured with a Leica SP8 confocal microscope (Leica Microsystems, Wetzlar, Germany), using a 20x lens with a numerical aperture of 1.0. The images were cut in 2.52 *μ*m for all embryos analyzed as well as the other settings of the microscope. The fluorescent images recorded were analyzed using Adobe Photoshop CC 2017 version 18.0.0 (Adobe Systems Incorporated, San Jose, CA, USA). The image resolution was 72 pixels, and the intensity scale ranged from zero, indicating the absence of fluorescence, to 255, indicating the maximum recordable fluorescence. For GSH assessment, an epifluorescence microscope was used (Axioplan 2, Zeiss®). The fluorescence intensities expressed were analyzed in arbitrary fluorescence units (pixels).

### 2.5. Gene Expression Analysis by Quantitative Real-Time Polymerase Chain Reaction (qRT-PCR)

Transcription levels of four genes related to the metabolism of ROS (catalase (CAT), manganese superoxide dismutase (SOD2), glutathione peroxidase 4 (GPX4), and peroxiredoxin 3 (PRDX3)) and four genes involved in the apoptosis process (caspase 3 (CASP3), caspase 8 (CASP8), B-cell lymphoma 2 (BCL2L1), and Bcl2-associated X protein (BAX protein)) were quantified.

Three pools of 18 embryos were used for each treatment. The total RNA was extracted using the RNeasy Plus Micro Kit (Qiagen®, Hilden, Germany), following the manufacturer's instructions (with minor modifications). The total volume of RNA samples was incubated directly with 1 U DNase I (Invitrogen®/Life Technologies, *ΝY*, USA) at 37°C for 30 min. The cDNA synthesis was performed using SuperScript III (200 U/*μ*L, Invitrogen) and Oligo-dT primer (0.5 *μ*g/*μ*L, Invitrogen) in a final volume of 28 *μ*L. The reactions were performed at 65°C for 5 min and at 42°C for 60 min, followed by inactivation of the enzyme at 70°C for 15 min.

The qRT-PCR was performed in a 7500 Fast Real-Time PCR System (Applied Biosystems, Foster City, CA, USA) using three independent biological replicas. The specificity of each PCR product was determined by analyzing the melting curve and the amplicon size in agarose gel. The qRT-PCR was performed using Fast Sybr Green Master Mix (Applied Biosystems). The reactions were optimized to promote the efficiency of maximum amplification for each gene (90-100%). Each sample was analyzed in triplicate, and the specificity of each PCR product was determined by analyzing the melting curve and amplicon size in agarose gel. The reactions were carried out in a final volume of 25 *μ*L cDNA corresponding to 0.9 D7 embryos. For each gene sample and every plate of runs, a negative control was present, in all biological replicates. The conditions of the qRT-PCR cycles were 95°C for 5 min, followed by 50 cycles of denaturation at 95°C for 15 s and annealing at 60°C for 1 min. The name, sequence, and concentration of the primer and the size of the amplicon, of each gene, are listed in Table 1.

The expression levels (based on the cycle threshold (Ct) values, from the three biological replicates) of the three constituent genes, glyceraldehyde-3-phosphate dehydrogenase (GAPDH), *β*-actin (ACTB), and peptidylprolyl isomerase A (PPIA), were submitted to the GeNorm analysis program, which showed GAPDH as the most stable gene. This gene was used as a reference for database normalization. The relative expression (Ct values) for each gene was calculated using the *ΔΔ*Ct method with efficiency correction by the Pfaffl method [38].

### 2.6. Cryopreservation and Thawing of Expanded Blastocysts (BX)

After 168 h of fertilization (D7), the embryos were evaluated in terms of their morphology in order to determine their development stage according to the International Embryo Technology Society (IETS) manual. Only grade 1 and 2 expanded blastocysts [39] of each group were cryopreserved by the direct transfer (DT) method described by Sanches et al. [40]. In summary, the BX were exposed to a freezing solution (1.5 M ethylene glycol) for 10 min at room temperature (25°C). After this stabilization period, 5 embryos from each group were placed in 0.25 mL straws and transferred to the freezing equipment (Freeze Control-CryoLogic®, Blackburn, Victoria, Australia), previously stabilized at -6°C. The freezing device was set to a freezing rate of 0.5°C per min, from −6 to −35°C. After reaching −35°C, the straws were immersed in liquid nitrogen. After immersing in liquid nitrogen (5 min), the embryos were thawed. Each straw was removed from the liquid nitrogen and kept in air for 10 s and in a water bath at 35°C for 30 s. After thawing, the straws were dried with a paper towel and agitated to allow the mixture of the solutions inside the straw. Embryos were released from the straws, were transferred back to the culture drop corresponding to the experimental group, and were cultured for an additional 48 h. The reexpansion and hatching were assessed at 12, 24, 36, and 48 h postthawing. The control groups for both treatments were kept on the bench in a holding medium during the freeze-thaw process.

### 2.7. Experimental Design and Statistical Analysis

#### 2.7.1. Experiment 1: Quantification of Total Phenolic Compounds and Antioxidant Activity of Dried Leaves from Brazilian *Cerrado* Plants

In this experiment, five native *cerrado* plants, namely, cashew, cagaita, araticum, murici, and jenipapo, were tested. The objective was to determine which of the ethanolic extracts had the highest total phenolic compound content (Folin-Ciocalteu method) as well as the highest antioxidant activity (ABTS method).

#### 2.7.2. Experiment 2: Effect of Different Concentrations of Ethanolic Extracts of Dried Leaves of Brazilian *Cerrado* Plants on the *In Vitro* Production of Bovine Embryos

In experiment 1, the ethanolic extracts of cagaita and murici dried leaves had the highest antioxidant activity and total phenolic compound content values (Table 2). Therefore, they were chosen to be used in experiment 2.

The objective of this experiment was to determine the best concentration of cagaita and murici extracts to be added to the culture media to improve embryonic quality. Due to the large number of oocytes needed per replicate/day, the cagaita and murici extracts were not tested together, but in two separate assays. In each assay, at the end of IVF, presumptive zygotes were randomly distributed into four groups that were exposed to various extract concentrations. The following groups were treated with the cagaita extract: control (embryos cultured in conventional culture media–SOF); a group in which SOF was supplemented with 0.01 mg/mL of the cagaita extract (Cag0.01); a group with 0.1 mg/mL of the cagaita extract (Cag0.1); and a group with 1 mg/mL of the cagaita extract (Cag1). The following groups were treated with the murici extract: control (embryos cultured in conventional culture media–SOF); a group in which SOF was supplemented with 0.01 mg/mL of the murici extract (Mur0.01); a group with 0.1 mg/mL of the murici extract (Mur0.1); and a group with 1 mg/mL of the murici extract (Mur1). The total extract added to the SOF was 1% (*v*/*v*). The embryos were evaluated for cleavage and blastocyst rates, embryonic development, total number of cells, and percentage of apoptotic cells.

To evaluate if the time of culture and the presence of embryos would affect the antioxidant activity of the different plant extract concentrations, the culture media were also analyzed. A sample of each concentration/extract was collected at the time of the culture drop preparation, which was considered the medium on D0. Then, for each drop/extract/concentration, an additional drop was placed in the dish and was kept for seven days without any embryo. All samples of the media were analyzed for antioxidant activity by the ABTS method.

#### 2.7.3. Experiment 3: Effect of Cagaita and Murici Ethanolic Extracts on the Oxidative Stress of Blastocysts Produced *In Vitro*

The objective of this experiment was to evaluate the influence of the extracts in regulating embryonic oxidative stress. For this experiment, the best murici (Mur0.01) and cagaita (Cag0.01) extract concentrations obtained in experiment 2 were selected. Besides the SOF media (supplemented with murici and cagaita) and the control (SOF with no extract) treatments, an additional group was added. This last group was referred to as low O_2_ tension (G5%), in which presumptive zygotes were cultured in SOF with no extracts under a 5% O_2_ atmosphere. The rationale behind the addition of this group was to assess whether the level of oxidative stress in embryos cultured with plant extracts for seven days would be the same as that in embryos cultured with no plant extracts but at a lower O_2_ concentration (5% O_2_). After the culture period ended, the embryos were used for the quantification of ROS, GSH, and transcription levels of genes related to oxidative stress and to apoptosis in expanded blastocysts.

#### 2.7.4. Experiment 4: Effect of Cagaita and Murici Ethanolic Extracts on the Cryotolerance of Expanded Blastocysts Produced *In Vitro*

This experiment is aimed at evaluating the effect of cagaita and murici ethanolic extracts on the cryotolerance of embryos. Five treatments were proposed: fresh control, composed of expanded embryos, and four cryopreserved groups (the control group: consisting of embryos cultured in conventional culture media–SOF and frozen by the direct transfer (GDT) method; Cag0.01: group in which SOF was supplemented with 0.01 mg/mL of cagaita extract and frozen; Mur0.01: group in which SOF was supplemented with 0.01 mg/mL of murici extract and frozen; and G5%: group in which presumptive zygotes were cultured in SOF with no extracts under a 5% O_2_ atmosphere). The expanded embryos were frozen according to the protocol described by Sanches et al. [40]. After thawing, the embryos returned to the original medium and were cultured for an additional 48 h. The embryos were evaluated at 12, 24, 36, and 48 h postthawing for reexpansion, hatching, and degeneration. In another experiment, identical to this, ROS and apoptotic rates were assessed 12 h postthawing in expanded blastocysts.

### 2.8. Statistical Analysis

Antioxidant activity data obtained by the ABTS method and the amount of total phenols obtained by the Folin-Ciocalteu method were analyzed by linear regression. Cleavage and blastocysts on D6 and D7, embryonic development, reexpansion, hatching, degeneration and apoptosis, intracellular levels of ROS and GSH, and relative abundance of mRNA were evaluated by analysis of variance (ANOVA) (parametric data) and the Kruskal-Wallis test (nonparametric data). The test for average comparison was carried out using Tukey's test. The 5% (*p* < 0.05) significance level was used for all analyses. All statistical analyses were performed using the GraphPad Prism 6 software (La Jolla, CA, USA).

## 3. Results

### 3.1. Total Phenolic Compounds and Antioxidant Activity of Brazilian *Cerrado* Plants

Among the five plant extracts that were evaluated, the extracts with the highest antioxidant activity values were the murici and the cagaita (*p* < 0.05) (Table 2). Regarding the total phenolic compound content, the murici ethanolic extract had the highest concentration (*p* < 0.05). Based on antioxidant activity, murici and cagaita were selected to be tested in other experiments.

### 3.2. Effect of Different Concentrations of the Ethanolic Extract of Dried Cagaita and Murici Leaves on the *In Vitro* Production of Bovine Embryos

To evaluate the best dry cagaita extract concentration, 390 embryos from 902 oocytes (43.2%) were produced, in seven replicates (Table 3). The cleavage was similar for all groups. However, Cag1 (0.01 mg/mL) showed a lower rate (*p* < 0.05) of blastocysts on D6 and D7 when compared to Cag0.01.

The total cell number of expanded blastocysts was similar among the groups tested (*p* > 0.05). Nevertheless, when the apoptotic cell percentage was assessed, Cag0.01 had a lower apoptotic cell rate (2.8%) than the other groups. The Cag0.1 and Cag1 groups had similar apoptotic cell rates (Figure 1, Table 3).

Regarding embryonic development on D6, all treatments showed that embryos were at the same stage of development (Table 4). However, Cag1 had lower blastocyst (BL) and BX rates compared with Cag0.01 on D7 (*p* < 0.05).

When murici was evaluated in the IVP, 684 oocytes that produced 196 embryos were used (Table 3). The cleavage and the embryonic production on D6 and D7 were similar in all groups tested (*p* > 0.05), except for Mur1 that did not even cleave. The average total cell number was higher (*p* < 0.05) in the control group (142.6 ± 33.8) than in Mur0.1 (114.2 ± 24).

The embryonic development was similar for groups Control, Mur0.01, and Mur 0.1, as the percentage of embryos reaching the BL and BX stage on D6 and D7 was similar in the control groups as well as in Mur0.1 and Mur0.01 (Table 4). Mur1 had a deleterious effect on embryonic development from the beginning, considering that there was no cleavage; therefore, no embryos were formed.

**Figure 2:**
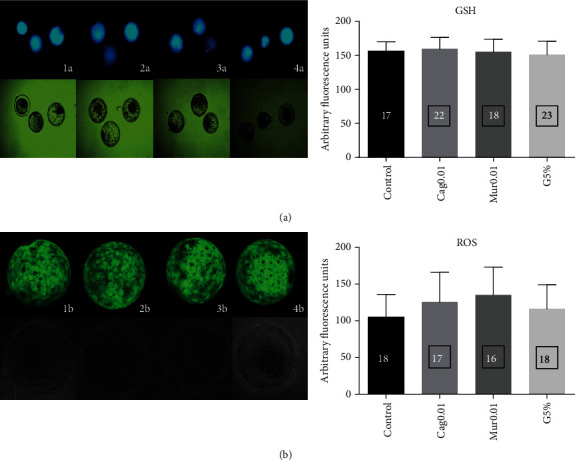
Intracellular GSH levels of bovine expanded blastocysts evaluated with CellTracker Blue (a) and ROS evaluated with H_2_DCFDA (b) under different 20% O_2_ culture systems: (1) control: without ethanolic extract supplementation; (2) Cag0.01: with the addition of 0.01 mg/mL of the dried cagaita leaf extract; (3) Mur0.01: with the addition of 0.01 mg/mL of the dried murici leaf extract; and (4) G5%: embryo culture under 5% O_2_.

The antioxidant activity (Figure 2—Supplementary files) of the cagaita ethanolic extract was not affected by the time of culture or by the presence of embryos. However, the murici ethanolic extract had lower antioxidant activity on D7, when it was used in a concentration of 1 mg/mL and in the presence of embryos.

### 3.3. Effect of Cagaita and Murici Ethanolic Extracts on the Oxidative Stress of Blastocysts Produced *In Vitro*

A total of 893 embryos from 2135 oocytes (41.8%) were produced in this experiment in eight replicates. All embryonic production parameters were similar among the groups (Table 5).

The stage of development on D6 and D7 was similar for all groups (*p* > 0.05); therefore, the ethanolic extracts did not affect the embryonic development (Table 1—Supplementary files).

The fluorescent emission of ROS (Figure 2) was similar (*p* > 0.05) among the control (105.24 ± 26.04 pixels), Cag0.01 (125.92 ± 31.82 pixels), Mur0.01 (135.25 ± 29.05 pixels), and G5% (116.05 ± 27.51 pixels) groups. Similar results were observed in the evaluation of the GSH fluorescent emission levels (Figure 2) among the control (156.36 ± 11.39 pixels), Cag0.01 (159.98 ± 10.89 pixels), Mur0.01 (155.36 ± 14.07 pixels), and G5% (151.37 ± 17.45 pixels) groups.

**Figure 3:**
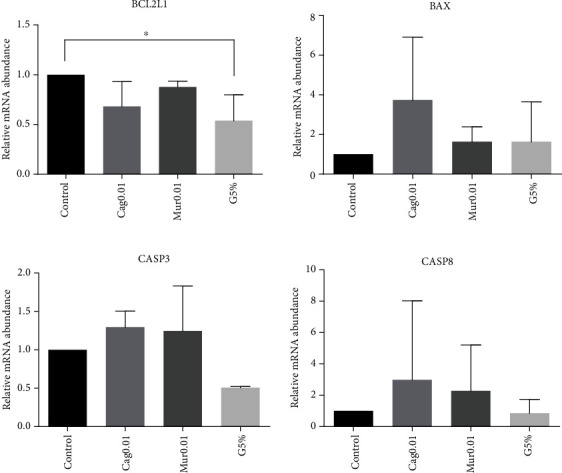
Relative mRNA abundance of genes involved in the apoptosis process. BCL2L1, BAX, CASP3, and CASP8 analyses by qRT-PCR in expanded blastocysts cultured under 20% O_2_ (control group, supplemented with 0.01 mg/mL ethanolic extract of dried cagaita (Cag0.01) or murici (Mur0.01) leaves) or under 5% O_2_ (G5%). Mean ± standarddeviation of three biological replicates. Data were normalized using the formula *ΔΔ*CT [38] and GAPDH as an endogenous control gene. ^∗^*p* value = 0.058 by *t*-tests.

Regarding gene expression, the results showed that the transcription levels of three out of the four genes related to apoptosis, BAX, CASP3, and CASP8, were similar (*p* > 0.05) among the groups (Figure 3). However, when analyzing BCL2L1, the number of transcripts was higher in the control than in the G5% group (*p* = 0.058).

**Figure 4:**
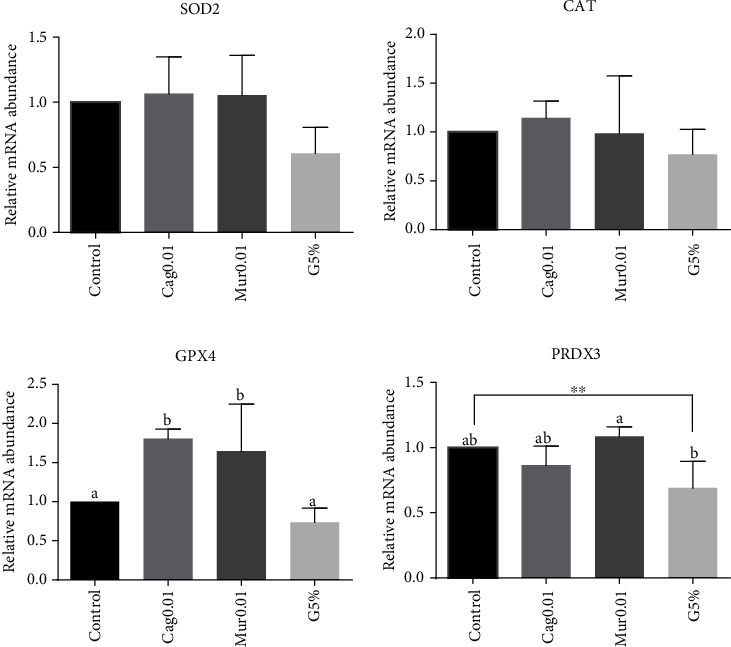
Relative mRNA abundance of genes involved in the reactive oxygen species pathways in the apoptosis process. SOD2, CAT, GPX4, and PRDX3 analyses by qRT-PCR in expanded blastocysts cultured under 20% O_2_ (control group, supplemented with 0.01 mg/mL ethanolic extract of dried cagaita (Cag0.01) or murici (Mur0.01) leaves) or under 5% O_2_ (G5%). Mean ± standarddeviation of three biological replicates. Data were normalized using the formula *ΔΔ*CT [38] and GAPDH as an endogenous control gene. Bars with different superscript letters indicate significant differences (*p* < 0.05) by *t*-tests. ^∗∗^*p* value = 0.07.

Concerning the genes involved in the metabolism of ROS, GPX4 was expressed (*p* < 0.05) at a higher level in the groups cultured with cagaita and murici than in the groups without these extracts (control and G5%). PRDX3 was expressed at a higher level (*p* < 0.05) in Mur0.01 than in G5% (Figure 4). Genes from the enzymatic antioxidant system herein analyzed (SOD2 and CAT) were similar among the treatments (*p* > 0.05).

### 3.4. Effect of Ethanolic Extracts of Dried Cagaita and Murici Leaves on the Cryotolerance of Expanded Blastocysts Produced *In Vitro*

**Figure 5:**
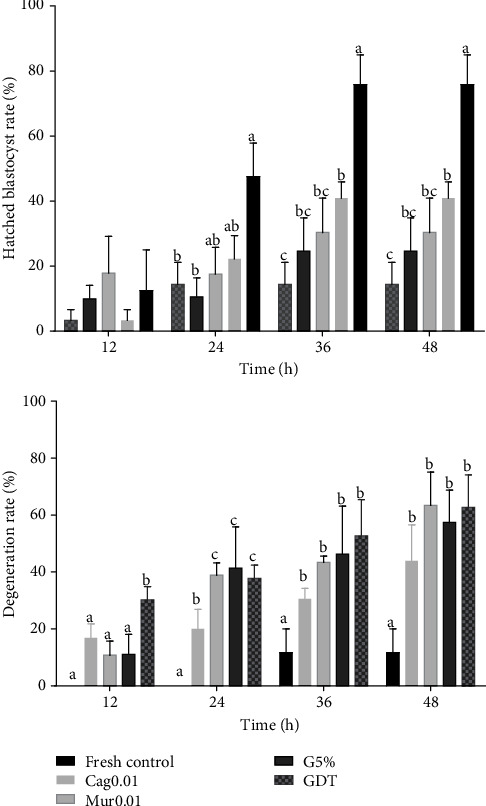
Hatching and degeneration rates 12, 24, 36, and 48 h after thawing of expanded blastocysts cryopreserved by the direct transfer (DT) method under 20% O_2_ (GDT) supplemented with 0.01 mg/mL ethanolic extract of dried cagaita (Cag0.01) or murici (Mur0.01) leaves and under 5% O_2_ (G5%) and in the noncryopreserved group (fresh control). ^a,b,c^Different superscript letters indicate significant differences between treatments at a given time point (*p* < 0.05).

The rate of reexpansion was similar for all treatments (*p* > 0.05) at all time points. Nevertheless, differences in hatching rates among the groups were observed at 24 h where the fresh control group showed higher (*p* < 0.05) rates than the GDT and G5% groups. When the cryopreserved groups were evaluated 36 and 48 h postthawing, Cag0.01 had a higher hatching rate compared with GDT (*p* > 0.05). The other cryopreserved groups had similar rates (Figure 5).

At 12 h, the degeneration rate was higher in the GDT group than in the other treatments (*p* < 0.05). In the remaining time points (24, 36, and 48 h), the fresh control group showed the lowest degeneration rate. However, Cag0.01 had a lower degeneration rate among the cryopreserved groups 24 h after thawing. This behavior was not preserved in subsequent assessments (36 and 48 h), as is shown in Figure 5.

**Figure 6:**
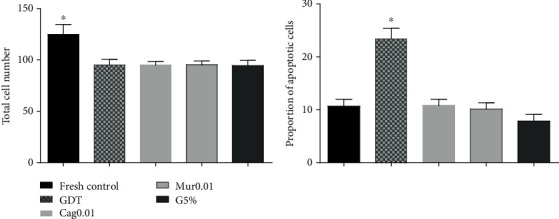
Total cell number and proportion of apoptotic cells in bovine expanded blastocysts 12 h after thawing of expanded blastocysts cryopreserved by the direct transfer (DT) method under 20% O_2_ (GDT) supplemented with 0.01 mg/mL ethanolic extract of dried cagaita (Cag0.01) or murici (Mur0.01) leaves and under 5% O_2_ (G5%) and in the noncryopreserved group (fresh control). ^∗^Group differed from the others (*p* < 0.05).

One hundred thirty-four embryos were analyzed for the ROS production, apoptosis rate, and total number of cells. There was no difference (*p* > 0.05) in ROS production among treatments. The control group had a higher number of cells than the other groups. However, the proportion of apoptotic cells was higher in GDT (*p* > 0.05), as is shown in Figure 6.

## 4. Discussion

The search for natural compounds with high antioxidant activity to be used as alternatives for the improvement of embryonic quality has been an issue of growing interest among research groups [19, 21, 41–47]. In this context, the *cerrado* biome, characterized by great biodiversity, becomes a potential source for obtaining these compounds. In fact, studies on the molecular characterization of *cerrado* plants have shown that they have high concentrations and a wide variety of phenolic compounds, with proven antioxidant, neuroprotective, anti-inflammatory, antiparasitic, and antifungal properties [27–30, 48, 49]. Considering this scenario, we hypothesized that IVP embryos supplemented with ethanolic extracts of *cerrado* plants would have improved quality and cryotolerance.

The plants used were chosen based on previous studies [29, 49–51], which have shown their antimicrobial, anti-inflammatory, and antifungal properties. The plant extracts were then evaluated for their antioxidant activity by the ABTS method, and the results showed a large variation among them. The use of the murici extract resulted in higher values than those reported by Silva et al. [52], when extracts of dried leaves of this plant harvested from the Pará state were analyzed for both parameters. On the other hand, the use of the cagaita leaf extract resulted in lower values compared with the total phenolic compound concentration and antioxidant activity results obtained by Takao et al. [27] who used leaves harvested from the São Paulo state. Such differences among different plant species are expected, as well as some variations in the concentrations and types of polyphenols within the same plant, depending on the biological material analyzed (leaves, fruits, and bark), the region where the plants are located, and the season [53]. Based on the results obtained in the first experiment, the murici and cagaita extracts were selected to be added in the *in vitro* system for bovine embryo production.

As there are no reports in the literature examining the use of these compounds for embryo production, it was necessary to establish the best concentrations before adding them to the culture media. We choose the standard concentration for crude ethanolic extracts (1 mg/mL) based on the previously tested antimicrobial activities of those plants [54–56]. Various concentrations from murici and cagaita were tested. The highest cagaita extract concentration (Cag1) tested was deleterious to the embryos. This effect was evident by the lower blastocyst rate and a delay in the embryonic development on D7 compared with the Cag0.01 group. This effect was exacerbated when murici extract was used at the same concentration of 1 mg/mL (Mur1), as it completely inhibited embryonic development. However, it is important to point out that the deleterious effect was only observed when high concentrations were used; as the extract concentration decreased, this effect was no longer detected.

It is well known that ROS acts as a second messenger in many metabolic systems that are vital to the cell, such as protein transport and transcriptional activity regulation [2, 57, 58]. However, while low ROS levels stimulate cell proliferation, high levels may result in cytotoxic action [1, 59]. Therefore, it is possible that the deleterious effect observed when high concentrations were used was a result of the high antioxidant activity of the extracts. This may have substantially decreased the production of ROS at the beginning of embryonic development, thus impairing intracellular signaling in the zygote. This hypothesis was reinforced by the analysis of the culture medium supplemented with 1 mg/mL of murici (Mur1), which showed high antioxidant activity, even after seven days of culture. The persistence of high antioxidant activity for seven days may have impaired embryonic development in Mur1 and, to a certain extent, the development of embryos in Mur0.1.

Conversely, the lower extract concentrations did not affect blastocyst rates and embryonic development but influenced the rate of apoptosis. In preimplantation embryos, in spite of apoptosis being considered a physiological process, morphological abnormalities in embryos could be correlated with a high incidence of apoptotic cells, according to Wang et al. [47]. The supplementation of 0.01 mg/mL of cagaita decreased the rate of apoptosis in relation to the other concentrations, which may be an indication of better quality. This dose-response effect, which shows toxicity in higher concentrations and improvements in embryo quality when cultured in lower concentrations, has been described previously using different extracts, like green tea and Asian native plants [44, 47, 60].

After choosing the most appropriate concentration (0.01 mg/mL of each extract), we evaluated the embryonic production and the intracellular levels of ROS and GSH. The presence of plant extracts in culture media or low O_2_ tension (G5%) had no effect on embryonic production and development. Similarly, the extracts had no effect on the ROS and GSH intracellular levels. Maybe its effects were undetected at the time that evaluation was performed. Surprisingly, cultures under low O_2_ tension did not affect either the ROS or GSH levels. According to Leite et al. [61] and others [9, 62, 63], the production of ROS in embryos cultured under low oxygen tension is lower compared to embryos cultured under atmospheric concentrations of O_2_. One explanation could be that the control group, as well as Cag0.01 and Mur0.01 cultured under 20% oxygen tension, was cocultured with cumulus cells. According to Corrêa et al. [64], the presence of cumulus cells contributes to minimizing deleterious effects caused by higher oxygen tension. Indeed, the transcription levels of ROS metabolism, which were similar among the groups, may not have contributed to the elimination of ROS from this group; it is known that ROS production is regulated by an enzymatic antioxidant system and a nonenzymatic system, such as glutathione [59].

Our results showed that supplementing embryos with cagaita and murici ethanolic extracts did not affect the ROS and GSH levels of expanded blastocysts. Results reported by Madrid Gaviria et al. [65] using 0.5 *μ*M of resveratrol during embryo culture also showed no changes in ROS and GSH levels in fresh embryos cultured compared to the control. In addition, Rocha-Frigoni et al. [66] found no changes in ROS levels of blastocysts cultured in the presence of 100 *μ*M of *β*-mercaptoethanol.

Several endogenous antioxidant enzymes have been directly involved in embryo ROS protection, such as enzymes from the superoxide dismutase family, catalase, and glutathione peroxidase [67]. The results of gene expression regarding cellular stress suggested that supplementing the culture medium with the murici and cagaita extracts had no effect on SOD2 and CAT transcription levels. However, the GPX4 transcription level was significantly higher in Cag0.01 and Mur0.01, which could offer better protection against membrane peroxidation, by their ability to reduce hydroperoxides in complex lipids [68]. Nevertheless, the action of this gene is not restricted to the metabolism of ROS. It also regulates cell apoptosis, as it can neutralize the activity of 12/15 lipoxygenase, an enzyme responsible for activating the apoptosis-inducing factor, an alternative pathway for apoptosis besides caspases [69]. This increase in the GPX4 transcription levels seems to have contributed to the decrease in the rate of apoptosis in the embryos cultured with cagaita extract, once the other apoptosis-inducing genes assessed (CASP3 and CASP8) were not differentially expressed among treatments. Also, the addition of these extracts did not change the transcription level of key genes in the regulatory mechanism of apoptosis, such as the proapoptotic BAX and the antiapoptotic BCL2L1, in expanded blastocysts. Some authors have already reported a similar behavior in these genes in embryos supplemented with resveratrol during *in vitro* culture [65, 70]. These results show a likely antiapoptotic action of ethanolic cagaita extract through the increased transcription levels of GPX4 and not by other metabolic pathways involved in the complex mechanism of apoptosis.

The cagaita crude extract contains an abundance of phytocompounds, such as sesquiterpenes, polyphenols compounds (catechin, quercetin, and epicatechin ellagic acid), anthocyanins, and carotenoids, among other bioactive substances [28, 31]. The cagaita's phytocompounds probably allow the redox status reversibility through catechol/quinone conversion and, thus, strengthen the antioxidant activity [71, 72]. However, this effect was not observed when the murici crude extract was used. It is possible that the bioactive compounds in the murici crude extract do not act independently, meaning that the response of embryos to that plant extract may be the result of synergistic, antagonistic, or other interactive effects among its biological components [73, 74], which could explain the absence of a correlation between evaluated genes and apoptosis.

In addition to the parameters evaluated, we also assessed the ability of the embryos to respond to cryopreservation. It is well accepted that cryotolerance is a highly reliable marker of embryonic quality [75–77]. In this regard, we clearly demonstrated a significant reduction in apoptosis and degeneration rates 12 h postthawing in embryos cultured in the presence of cagaita and murici extracts and under 5% O_2_. Indeed, the hatching rate at 48 h after thawing observed in Cag0.01, which was higher than GDT (cryopreserved group), indicates a positive effect of that extract, since the embryonic hatching ability is an indicator of quality [78, 79]. This increase in the hatching rate, caused by the addition of cagaita and murici extracts, is similar to that described by Zullo et al. [80], using 1 *μ*M of crocetin (carotenoid component of saffron). These authors suggested that the effect could be explained by the high antioxidant capacity, which protects the embryo from oxidative stress caused by the freeze-thaw process, by preventing lipid peroxidation, membrane injury, and structural damage. Conversely, the benefits of low oxygen tension during culture on embryonic quality have been described previously [81–83]. However, this positive cryotolerance effect is not always reported [84]. Our results showed an improvement in the cryotolerance of embryos cultured under low tension, but the benefits of the extract supplementation, especially cagaita at 0.01 mg/mL, proved to be more efficient than the others, in terms of both the degeneration and hatching rates.

## 5. Conclusion

In conclusion, the results of this study demonstrated that supplementation of the cagaita ethanolic extract of dry leaves improves IVP blastocysts' hatching rates and decreases apoptotic rates after thawing, suggesting that it may be used as an alternative to increase cryopreservation efficiency. To our knowledge, this is the first study to demonstrate the positive effects of ethanolic extracts of *cerrado* plants on cryotolerance of bovine blastocysts produced *in vitro*. However, further studies are required to evaluate other antioxidant and apoptotic pathways, as well as to assess the effect of embryos cultured with the extracts on pregnancy rates.

## Figures and Tables

**Table 1:** Specific primer information used for quantitative real-time PCR (qRT-PCR) gene fragment amplification.

Gene	Primer sequence	Amplicon size (pb)	Primer concentration (nM)	GenBank access number
GAPDH	F: GGC GTG AAC CAC GAG AAG TAT AA R: CCC TCC ACG ATG CCA AAG T	118	300	NM_001034034.2
SOD2	F: TTG CTG GAA GCC ATC AAA CGT GAC R: AAT CTG TAA GCG TCC CTG CTC CTT	135	300	NM_201527
CASP3	F: GCC CAG GAC TTT AGC AGT CA R: AAA TGT GAG CGC CTT TGT T	185	250	NM_001077840.1
CASP8	F: CAG AAC AGA TGG AAG CCT AT R: GGT TAG GAT GGT CAG AAT GT	209	250	NM_001045970.2
CAT	F: GAA TGA GGA GCA GAG GAA AC R: CTC CGA CCC TCA GAG ATT AG	241	300	NM_001035386.2
GPX4	F: CGC CGA GTG TGG TTT AC R: AGG TCC TTC TCT ATC ACC AG	315	300	NM_001346431.1
PRDX3	F: GGC AGG AAC TTT GAT GAG AT R: GTG TGT AGC GGA GGT ATT TC	205	300	NM_174643.1
BAX	F: TGC AGA GGA TGA TCG CAG CTG TG R: CCA ATG TCC AGC CCA TCA TGG TC	198	300	[85]
BCL2L1	F: GAG ATG CAG GTA TTG GTG AG R: GGT CAG TGT CTG GTC ATT TC	244	250	NM_001077486.2

F: primer forward; R: primer reverse. GAPDH: constitutive gene.

**Table 2:** Antioxidant activity and total phenolic compound (TPC) content of the ethanolic extract of dry *cerrado* plant leaves.

*Cerrado* plants	Antioxidant activity (*μ*M Trolox/g)^∗^	TPC (gallic acid/100 g)^∗∗^
Araticum (*Annona montana*)	190.6 ± 3.7^c^	192.1 ± 17.3^b^
Cagaita (*Eugenia dysenterica*)	729.7 ± 10.2^a^	201.1 ± 10.7^b^
Cajuzinho (*Anacardium humile*)	397.3 ± 4.2^b^	173.2 ± 11.9^b^
Jenipapo (*Genipa americana*)	89.2 ± 3.8^d^	111.7 ± 12.4^c^
Murici (*Byrsonima crassifólia*)	844.0 ± 9.7^a^	259.2 ± 13.8^a^

Legend: different letters in the same column differ from each other (*p* < 0.05). ^∗^ABTS radical inhibition of extracts is determined as a function of the Trolox pattern in *μ*M Trolox/g extract. ^∗∗^The evaluation was made by the Folin-Ciocalteu test. Results are expressed as mg gallic acid equivalent per 100 g extract.

**Table 3:** Bovine embryo production and apoptotic rates (percentage ± standarddeviation) with different concentrations (mg/mL) of ethanolic extract from dried cagaita and murici leaves.

	Cagaita ethanolic extracts (mg/mL)				Murici ethanolic extracts (mg/mL)			
	Control	Cag0.01	Cag0.1	Cag1	Control	Mur0.01	Mur0.1	Mur1
Total number of oocytes (*N*)	222	223	231	226	169	163	182	170
Cleavage rates (%)	80.6 ± 7.2	82.5 ± 9.4	81 ± 8.8	78.3 ± 5.3	80.5 ± 8.5	78.0 ± 11.1	81.9 ± 9.1	—
D6 blastocyst rates (%)	25.7 ± 8.4^ab^	34.1 ± 13.4^a^	25.5 ± 8.2^ab^	11.9 ± 9.0^b^	30.2 ± 9.1	31.9 ± 10.3	23.6 ± 15.4	—
D7 blastocyst rates (%)	45.5 ± 5.4^ab^	50.2 ± 14.4^a^	42.4 ± 6.0^ab^	35 ± 6.7^b^	40.8 ± 2.5	38.7 ± 8.1	35.2 ± 12.5	—
Embryo production (*N*)	101	112	98	79	69	63	64	—
Total number of BX—TUNEL (*N*)	38	37	39	21	23	24	27	—
Total cell number (*N*)	140.6 ± 29.9	139.6 ± 31.7	125.5 ± 33.4	120.2 ± 27.2	142.6 ± 33.8^a^	130.2 ± 34.5^ab^	114.2 ± 24^b^	—
Apoptotic cells/total cell number (%)	8.3 ± 2.4^a^	2.8 ± 2.1^c^	5.4 ± 2.6^b^	5 ± 2.9^b^	6.0 ± 3.1	5.7 ± 3.4	6.6 ± 2.8	—

Results from seven replicates. Control group: not supplemented with extracts. BX: expanded blastocyst; TUNEL: terminal deoxynucleotidyl transferase dUTP nick end labeling. Values with different superscript letters between lines, in the same extract, are significantly different (*p* < 0.05).

**Table 4:** Embryonic development 144 h (D6) and 168 h (D7) postinsemination (percentage ± standarddeviation) with different concentrations (mg/mL) of the ethanolic extract from dried cagaita and murici leaves.

Treatment	Oocytes (*N*)	Day 6				Day 7					
		EB (%)	BL (%)	BX (%)	Total	EB (%)	BL (%)	BX (%)	HB (%)	BE (%)	Total
Cagaita											
Control	222	63.3 ± 20.0	29.3 ± 16.3	7.4 ± 9.9	57	7.5 ± 7.8^a^	29.6 ± 11.0^ab^	57.0 ± 15.7^ab^	3.4 ± 4.7^a^	2.5 ± 3.3^a^	101
Cag0.01	223	46.8 ± 16.8	38.5 ± 10.7	14.8 ± 11.6	76	8.1 ± 8.1^a^	20.9 ± 10.9^b^	67.5 ± 11.8^a^	1.3 ± 2.2^a^	2.2 ± 2.9^a^	112
Cag0.1	231	41.6 ± 11.4	47.8 ± 11.7	10.6 ± 11.1	59	6.3 ± 9.0^a^	23.7 ± 8.5^b^	57.4 ± 17.4^ab^	9.0 ± 3.1^a^	3.7 ± 3.7^a^	98
Cag1	226	54.2 ± 31.5	39.6 ± 27.1	6.3 ± 10.4	27	13.2 ± 14.6^a^	49.8 ± 15.0^a^	32.1 ± 17.4^b^	2.4 ± 3.2^a^	2.6 ± 3.4^a^	79
Murici											
Control	169	56.9 ± 20.5	40.4 ± 20.9	2.8 ± 4.6	51	9.7 ± 9.7	34.2 ± 10.1	56.1 ± 11.2	—	—	69
Mur0.01	163	49.3 ± 19.4	37.2 ± 22.9	13.5 ± 9.8	52	7.5 ± 7.5	31.4 ± 14.0	53.8 ± 10.9	7.2 ± 7.2	—	63
Mur0.1	182	48.3 ± 15.6	46.3 ± 15.8	5.4 ± 7.1	43	1.2 ± 2	49.6 ± 19.4	47.9 ± 19.5	1.3 ± 2.1	—	64
Mur1	170	—	—	—	—	—	—	—	—	—	—

Results from seven replicates. Control group: not supplemented with extracts. EB: early blastocyst; BL: blastocyst; BX: expanded blastocyst; HB: hatching blastocyst; BE: hatched blastocyst. Values with different superscript letters within the same column, in the same extract, are significantly different (*p* < 0.05).

**Table 5:** *In vitro* embryo production rates (percentage ± standarddeviation) under different 20% O_2_ culture systems^1^.

	Control	Cag0.01	Mur0.01	G5%
Total number of oocytes	547	561	544	483
Cleavage rates	87 ± 8.1	89 ± 7.3	89 ± 8.3	88 ± 9.5
D6 blastocyst rates	26.5 ± 12.2	23.9 ± 10.3	27.2 ± 12.9	26.7 ± 16
D7 blastocyst rates	42.7 ± 6.2	42.9 ± 6.2	41.1 ± 5.4	40.1 ± 8.4

^1^Control: without ethanolic extract supplementation; Cag0.01: with the addition of 0.01 mg/mL of the dried cagaita leaf extract; Mur0.01: with the addition of 0.01 mg/mL of the dried murici leaf extract; G5% group: under 5% O_2_. Values with different superscript letters, in the same column, are significantly different (*p* < 0.05). Results from eight replicates.

## Data Availability

All data are provided in full in the results section of this paper.

## Supplementary Materials

Supplementary Materials Figure 1—Supplementary files: Trolox absorbance standard curve in micromoles per liter. Figure 2—Supplementary files: antioxidant activity of ethanolic extracts of dried cagaita and murici leaves by the ABTS method at two different time points during the *in vitro* culture. D0: beginning of zygote culture; D7: 168 h postinsemination; D7 incubator control: culture medium with the extract but without embryos. Table 1—Supplementary files: embryonic development 144 h (D6) and 168 h (D7) postinsemination (percentage ± standarddeviation) under different 20% O_2_ culture systems (control: without ethanolic extract supplementation; Cag0.01: with the addition of 0.01 mg/mL of the dried cagaita leaf extract; and Mur0.01: with the addition of 0.01 mg/mL of the dried murici leaf extract) and under 5% O_2_ (G5% group).
