# Supplementary material for: Ethanolic Extract of Dried Leaves from the Cerrado Biome Increases the Cryotolerance of Bovine Embryos Produced In Vitro
Source: Oxid Med Cell Longev. 2020 Nov 22;2020:6046013. doi: 10.1155/2020/6046013 (PMC7704130; doi:10.1155/2020/6046013)
Supplement: Supplementary Materials — Figure 1—Supplementary files: Trolox absorbance standard curve in micromoles per liter. Figure 2—Supplementary files: antioxidant activity of ethanolic extracts of dried cagaita and murici leaves by the ABTS method at two different time points during the in vitro culture. D0: beginning of zygote culture; D7: 168 h postinsemination; D7 incubator control: culture medium with the extract but without embryos. Table 1—Supplementary files: embryonic development 144 h (D6) and 168 h (D7) postinsemination (percentage ± standarddeviation) under different 20% O2 culture systems (control: without ethanolic extract supplementation; Cag0.01: with the addition of 0.01 mg/mL of the dried cagaita leaf extract; and Mur0.01: with the addition of 0.01 mg/mL of the dried murici leaf extract) and under 5% O2 (G5% group). [file 6046013.f1.docx]

The detailed description follows below:

Figure 1— Supplementary files: Trolox absorbance standard curve in micromoles per liter.

Figure 2 — Supplementary files: Antioxidant activity of ethanolic extracts of dried cagaita and murici leaves by the ABTS method at two different time points during the *in vitro* culture. D0: Beginning of zygote culture; D7: 168 h post insemination; D7 incubator control: culture medium with extract, but without embryos.

Table 1 — Supplementary files: Embryonic development 144 (D6) and 168 (D7) h post insemination (percentage ± standard deviation) under different 20% O_2_ culture systems (control: without ethanolic extract supplementation; Cag0.01: with the addition of 0.01 mg/mL of dried cagaita leaf extract; Mur0.01: with the addition of 0.01 mg/mL of dried murici leaf extract) and under 5% O_2_(G5% group).

**SUPPLEMENTARY FILES**


Figure 1— Supplementary files: Trolox absorbance standard curve in micromoles per liter.

Figure 2 — Supplementary files: Antioxidant activity of ethanolic extracts of dried cagaita and murici leaves by the ABTS method at two different time points during the *in vitro* culture. D0: Beginning of zygote culture; D7: 168 h post insemination; D7 incubator control: culture medium with extract, but without embryos.

* The 1 mg/mL group on D7 differs (p<0.05) from the other groups evaluated on D7.

Table 1 — Supplementary files: Embryonic development 144 (D6) and 168 (D7) h post insemination (percentage ± standard deviation) under different 20% O_2_ culture systems (control: without ethanolic extract supplementation; Cag0.01: with the addition of 0.01 mg/mL of dried cagaita leaf extract; Mur0.01: with the addition of 0.01 mg/mL of dried murici leaf extract) and under 5% O_2_ (G5% group).

| Treatment | Day 6 |  |  |  |  | Day 7 |  |  |  |  |  |
| --- | --- | --- | --- | --- | --- | --- | --- | --- | --- | --- | --- |
|  | EB % | BL % | BX % | Total |  | EB % | BL % | BX % | HB % | BE % | Total |
| Control | 49.3 ± 22.4 | 33.7 ± 19.2 | 17.0 ± 14.2 | 145 |  | 11.7 ± 9.8 | 31.0 ± 8.0 | 52.6 ± 16.0 | 3.8 ± 3.3 | 1.0 ± 1.6 | 234 |
| Cag0.01 | 45.9 ± 24.7 | 24.8 ± 15.1 | 29.3 ± 21.5 | 134 |  | 14.4 ± 7.7 | 29.0 ± 6.1 | 50.6 ± 10.4 | 5.6 ± 5.9 | 0.4 ± 0.7 | 241 |
| Mur0.01 | 49.3 ± 16.4 | 29.5 ± 8.2 | 21.2 ± 15.2 | 148 |  | 14.6 ± 10.3 | 26.5 ± 9.1 | 51.7 ± 7.6 | 6.1 ± 6.9 | 1.1 ± 1.9 | 224 |
| G5% | 43.1 ± 23.3 | 38.1 ± 14.4 | 18.8 ± 16.5 | 129 |  | 13.3 ± 11.1 | 32.6 ± 6.1 | 48.5 ± 14.1 | 5.2 ± 4.5 | 0.5 ± 0.8 | 194 |

Legend: Different letters in the same column differ from each other (p<0.05).

Control group: not supplemented with extracts.

EB: early blastocyst; BL: blastocyst; BX: expanded blastocyst; HB: hatching blastocyst; BE: hatched blastocyst.
